# Effectiveness of B Vitamins and Their Interactions with Aspirin in Improving Cognitive Functioning in Older People with Mild Cognitive Impairment: Pooled Post-Hoc Analyses of Two Randomized Trials

**DOI:** 10.1007/s12603-021-1708-1

**Published:** 2021-11-24

**Authors:** Y. Wu, A.D. Smith, H. Refsum, Timothy Kwok

**Affiliations:** 1 Department of Medicine and Therapeutics, The Chinese University of Hong Kong, Prince of Wales Hospital, Shatin, Hong Kong SAR, China; 2 Oxford Project to Investigate Memory and Ageing (OPTIMA), Department of Pharmacology, University of Oxford, Oxford, UK; 3 Department of Nutrition, Institute of Basic Medical Sciences, University of Oslo, Oslo, Norway

**Keywords:** Folic acid, vitamin B12, mild cognitive impairment, randomized trial, aspirin

## Abstract

**Background and Objectives:**

A randomized placebo-controlled trial found a significant negative interaction between aspirin and B vitamins in cognitive functioning in older people with mild cognitive impairment (MCI). To validate this finding, we pooled data of this trial with that of a similar B-vitamin trial (VITACOG) to examine the effectiveness of B vitamins and their interactions with aspirin in improving global cognitive functioning and slowing brain atrophy in older people with MCI.

**Design:**

Pooled post-hoc analyses of two randomized placebo-controlled trials.

**Participants:**

In total, 545 older people with MCI were included in the study.

**Intervention:**

Placebo or B-vitamin supplements (vitamin B12, folic acid with or without vitamin B6) for 24 months.

**Measurements:**

The primary outcome was the Clinical Dementia Rating scale-global score (CDR-global). The secondary outcomes were CDR-sum of box score (CDR-SOB), memory Z-score, executive function Z-score, and whole brain atrophy rate.

**Results:**

71 (26.2%) and 83 (30.3%) subjects in the active and placebo group respectively were aspirin users. Overall, B vitamins reduced whole brain atrophy rate significantly (P = 0.003), but did not have significant effect on CDR-global, CDR-SOB, memory and executive function. Aspirin use had significant negative interaction effects on B vitamins in CDR-global and CDR-SOB (Beta = 0.993, P = 0.038, and Beta = 0.583, P = 0.009, respectively), but not in memory or executive function Z-scores. Among aspirin non-users, B-vitamin group subjects had more favourable changes in CDR-global and CDR-SOB (P = 0.019 and 0.057, respectively). B vitamins significantly slowed brain atrophy in aspirin non-users (P = 0.001), but not in aspirin users, though the interaction term was not significant (Beta = 0.192, P = 0.276).

**Conclusion:**

In older people with MCI, B vitamins had significantly favourable effects on global cognitive functioning and whole brain atrophy rate in those who were not taking aspirin, but not in aspirin users.

## Introduction

**O**lder people with mild cognitive impairment (MCI) are at risk of dementia and have higher brain atrophy rate (1, 2, 3, 4). Elevated serum homocysteine is associated with MCI, dementia and brain atrophy (5). Suboptimal status of vitamin B12 and folate leads to elevation of serum homocysteine and supplementation of these vitamins with or without vitamin B6 can reduce serum homocysteine (6, 7, 8, 9). There have been randomized trials of B vitamins to improve cognitive function in older people with or without MCI or Alzheimer's disease (AD), showing inconsistent results (8, 10, 11, 12, 13, 14, 15, 16). The interpretation of these trials has been discussed (5, 17, 18).

The VITACOG trial in the United Kingdom (UK) was one of the few well designed trials which showed benefits of B vitamins (vitamin B12, folic acid and vitamin B6) in older people with MCI (7, 8). In this trial, there was a significant reduction of whole brain atrophy rate over 24 months with B vitamins (7). At the same time, there was a modest improvement in executive function (8). In the subgroup with high homocysteine (≤13 µmol/L) at baseline, B-vitamin treatment led to slowing of decline in episodic memory, semantic memory, Mini-Mental State Examination and Clinical Dementia Rating (CDR) [8] and a marked reduction in atrophy rate of key brain regions like the medial temporal lobe (19). A similar trial in Hong Kong (HK) using a lower dose folic acid (400 µg) and without vitamin B6 showed no benefit of B vitamins on cognitive function (9). More importantly, aspirin was found to have a significant negative interaction effect on B vitamins in cognitive functioning (9). Notably, in the VITACOG trial, aspirin use was also incidentally found to have a borderline significant negative interaction (P = 0.052) with B vitamins in slowing whole brain atrophy rate (7).

As both trials had similar design and used the CDR scale as an outcome, we pooled the two trial data sets to examine the effects of B vitamins on global cognitive functioning in older people with MCI. The potential interaction effects of aspirin in cognitive functioning and brain atrophy were specifically examined for.

## Subjects and methods

This retrospective analysis used pooled data from two randomized placebo-controlled trials: the VITACOG trial in the UK (Controlled-Trials.com, ISRCTN94410159) and the HK trial (Centre for Clinical Research and Biostatistics (CCRB) Clinical Trials Registry, CUHK_CCT00373) (7, 8, 9). The similarities in the design of the VITACOG and the HK trials justified pooling of the data: 1) participants in the UK and HK trials had MCI defined by Petersen's criteria (20, 21) and had comparable average age (76.8 and 77.4 respectively); 2) both trials randomized subjects in 1:1 to the active or placebo group; 3) the duration was 24 months in both trials; 4) both trials examined the effect of B-vitamin supplementation on cognitive functioning using the CDR scale and shared common cognitive test data for memory function; 5) both trials had volumetric brain MRI data at baseline and year two to estimate whole brain atrophy rate, the MRI scans were both carried out on a 1.5T MRI system with Tl-weighted acquisition and the same fully automated, quantitative method, SIENA, was used to derive the rate of whole brain atrophy per year, as described in detail in the original publications (7, 9). On the other hand, the VITACOG trial used a single tablet containing cyanocobalamin 500 µg, folic acid 800 µg and vitamin B6 20 mg once daily, while the HK trial used methylcobalamin 500 µg tablet and folic acid 400 µg tablet once daily. The cognitive outcomes were repeated once at month 24 in the UK trial, while they were repeated at month 12 and 24 in the HK trial. Both trials have been described in detail elsewhere (7, 9), and were carried out according to the principles of the Declaration of Helsinki and approved by local ethics committees.

The primary outcome was the CDR scale for global cognitive functioning (CDR-global) which is determined by an algorithm of 6 domain scores and ranged from 0–3 (9). CDR-sum of boxes score (CDR-SOB), ranging from 0–18, was obtained by summing each of the domain scores (9). The Category Fluency Test was used to measure executive function (and semantic memory) in both trials. Episodic memory was assessed by Hopkins Verbal Learning Test-delayed recall (HVLT-DR) in the VITACOG trial and International Shopping List Test (ISLT) in the HK trial (8, 9). Z-score of each subject was calculated according to the baseline mean and standard deviation (SD) of the respective trial population, with higher scores indicating better performance (9, 22). In a subgroup of 262 subjects with complete cranial MRI scans, whole brain atrophy rate (% per year) was calculated using SIENA package by estimating percentage brain volume change between baseline and follow-up (23). Change (Δ) in cognitive function was taken as the score at month 24 minus baseline score. ΔCDR-global categorized into decreased (ΔCDR-global < 0), unchanged (= 0) and increased (> 0) was the primary outcome. Annual whole brain atrophy rate, ΔCDR-SOB, Δexecutive function and Δmemory were the secondary outcomes.

Blood was taken after an overnight fast in HK trial and without fasting in VITACOG trial. The archived serum homocysteine and creatinine in the HK trial was analyzed by the same laboratory as in the VITACOG trial (7, 8). Serum sulphur amino acids, including homocysteine, were measured by liquid chromatography-tandem mass spectrometry as previously described (24). Serum folate, active vitamin B12 (holotranscobalamin, holoTC) and lipids were performed separately at Department of Pharmacology at University of Oxford in the UK and at Department of Chemical Pathology, Prince of Wales Hospital in HK (7, 8, 9).

### Statistical analyses

Data was presented as “mean ± SD”, “median (quartile 1 (Q1), Q3)” or “frequency (%)” as appropriate. Distributions of triglycerides, homocysteine, folate and vitamin B12 concentrations were all skewed, thus they were log-transformed to improve normality before performing further analysis. Baseline clinical characteristics between the two trial patients were compared using independent Student's t-test for continuous variables, or Chi-square test (χ2, or Fisher exact test) for categorical variables. Paired t-test was used to compare cognitive scores and serum homocysteine concentrations between baseline and 2 years follow-up in both groups. Pearson correlation or Spearman's rank correlation was applied for correlation analysis as appropriate. The B-vitamin effect on CDR-global, as well as secondary outcomes, were examined by general linear regression model, after adjusting for age, sex, education years, study site (from UK/HK), body mass index (BMI), smoking status, and history of diabetes and stroke.

The interaction effects of concomitant use of aspirin and B vitamins in cognitive functioning and cognitive test Z-scores was performed using general linear regression model, with adjustment for propensity scores of aspirin use and study site (25). The propensity score was calculated by logistic regression model based on age, sex, education years, BMI, smoking status, and history of diabetes and stroke. All statistical tests were two-sided and a p-value less than 0.05 was considered statistically significant (7, 8, 9, 22). All statistical analyses were performed with SPSS 24.0 for Windows (IBM Corp., Armonk, NY, USA).

## Results

**Figure 1:**
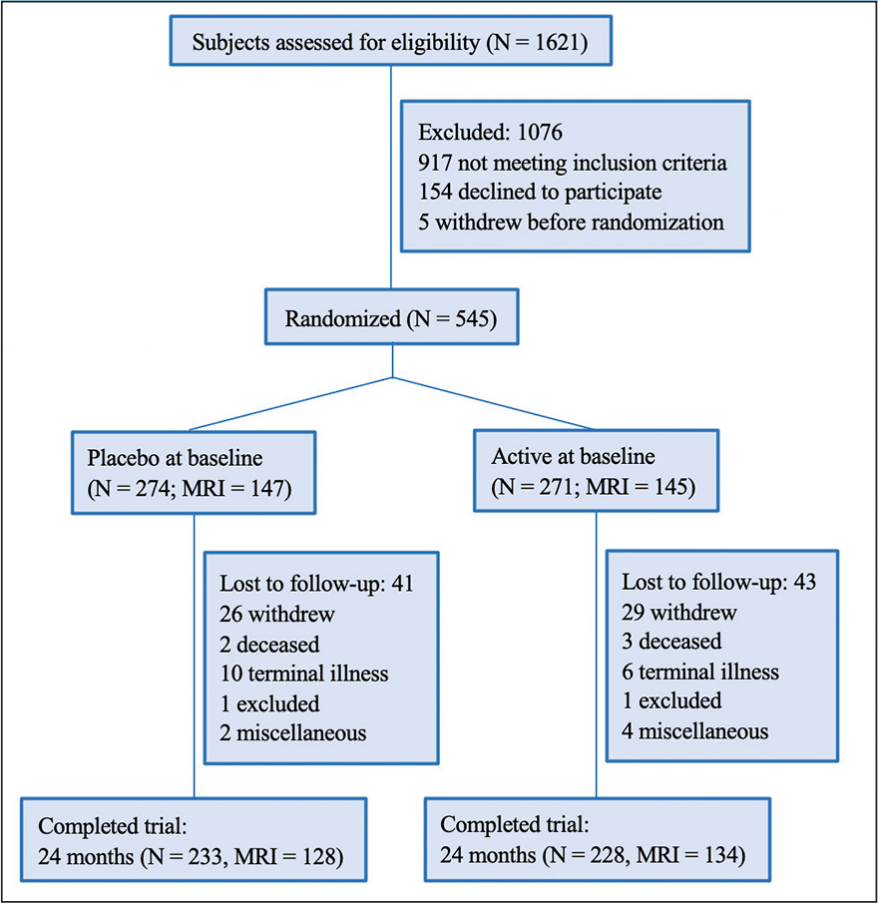
Flow of participants in the pooled VITACOG and HK trials

The population of this pooled analysis included MCI subjects from the VITACOG trial (N = 266) and from the HK trial (N = 279), giving a total sample size of 545 eligible participants. The participant flow was shown in Figure 1. The follow-up rates in the placebo group and the active group were 85.0% and 84.1% respectively. The follow-up rates of MRI scans in the two groups were 87.1% and 92.4% respectively.

The demographic and baseline characteristics of all participants are shown in Table 1. The placebo and active treatment groups were well-matched, except that the active group had better executive function at baseline (average Z-score 0.09 versus −0.09 in placebo group), and they were slightly younger (76.9 vs. 77.4). The differences in clinical characteristics of VITACOG and HK trial subjects were shown in Table S1. Compared with those in HK trial, subjects in the VITACOG trial had more women and longer duration of education and with more subjects having CDR-global = 0. VITACOG trial had more smokers, aspirin users (33.8% versus 22.9%) and higher body mass index, higher prevalence of stroke but a much lower prevalence of diabetes (5.3% versus 31.2%). They also had higher serum creatinine (mean 96.9 ± SD 16.6 versus 89.2°24.7 µmol/L) but lower homocysteine (median 11.4 (Q1 9.6, Q3 13.5) versus 16.6 (14.1, 19.8) µmol/L). These differences were well-adjusted in regression analyses. In total, 83 (30.3%) subjects in the placebo group and 71 (26.2%) in the active group were aspirin users, the usual dose being 80 mg once daily in both trials except that 9 HK trial subjects used 100 mg per day and another 5 used 160 mg per day. There were no significant differences between aspirin users and non-users except that aspirin users had higher prevalence of stroke, and lower total cholesterol. When compared with completers, the drop out subjects were older and more likely to have CDR-global score of 0 at baseline.

**Table 1:** Baseline characteristics of trial groups

	**Placebo (N = 274)**	**Active (N = 271)**	**P-value**
Age, yrs	77.4 ± 5.1	76.9 ± 5.1	0.265
Female, n (%)	147 (53.6%)	136 (50.2%)	0.418
Years of education, yrs	10.3 ± 6.0	10.7 ± 5.4	0.462
BMI, kg/m2	25.5 ± 3.8	25.2 ± 3.6	0.356
Ever smoking, n (%)	108 (39.6%)	100 (37.0%)	0.545
Aspirin user, n (%)	83 (30.3%)	71 (26.2%)	0.289
HBP, n (%)	190 (69.3%)	186 (68.6%)	0.858
DM, n (%)	54 (19.7%)	47 (17.3%)	0.477
Stroke, n (%)	25 (9.2%)	25 (9.3%)	0.967
Hb, g/dL	13.7 ± 1.3	13.7 ± 1.3	0.926
MCV, fL	91.5 ± 6.1	90.9 ± 6.8	0.239
TG #, g/L	1.2 (0.9, 1.7)	1.2 (0.9, 1.5)	0.577
TC, mmol/L	5.0 ± 1.2	5.1 ± 1.1	0.533
Cr, umol/L	93.7 ± 26.7	92.1 ± 19.1	0.388
Homocysteine #, µmol/L	13.9 (11.1, 17.3)	14.0 (11.3, 17.7)	0.798
Folate #, nmol/L	25.3 (18.0, 36.4)	25.5 (17.3, 33.9)	0.434
HoloTC #, pmol/L	74.9 (53.7, 102.0)	75.0 (54.0, 104.0)	0.981
CDR-global = 0, n (%)	60 (21.9%)	59 (21.8%)	0.971
CDR-SOB	1.1 ± 0.8	1.1 ± 0.9	0.990
Executive function Z-score	-0.09 ± 1.02	0.09 ± 0.97	0.045*
Memory Z-score	-0.06 ± 1.01	0.06 ± 0.99	0.170


Data were shown as “mean ± SD”, “median (Q1, Q3)” or “n (%)” as appropriate; # Use log-transformed data for comparison; * P-value < 0.05; BMI, body mass index; HBP, high blood pressure; DM, diabetes mellitus; Hb, hemoglobin; MCV, mean corpuscular volume; TG, triglycerides; TC, total cholesterol; Cr, creatinine; HoloTC, holotranscobalamin (active vitamin B12); CDR-global, Clinical Dementia Rating scale (CDR) global score; CDR-SOB, CDR sum of boxes score.

The comparison of the trial groups in changes in cognitive function and whole brain atrophy rates were shown in Table 2. No significant difference was found for the change of CDR-global, CDR-SOB, executive function and memory Z-scores over 24 months. Both placebo and active group subjects showed significantly worse CDR-SOB scores at follow-up (average ΔCDR-SOB = 0.23 and 0.14, P = 0.003 and 0.038, respectively). On the other hand, compared with the placebo group, B-vitamin supplementation reduced whole brain atrophy rate significantly (0.96% versus 0.73%, P = 0.003).

**Table 2:** The effects of B vitamins on cognitive function and whole brain atrophy rate in older people with MCI

	**Placebo**		**Active**		**P-value**
ΔCDR-global	↓	34 (15.7%)	↓	44 (20.4%)	0.132
	↔	157 (72.7%)	↔	155 (71.8%)	
	↑	25 (11.6%)	↑	17 (7.9%)	
ΔCDR-SOB	0.23 ± 1.10		0.14 ± 0.99		0.537
ΔExecutive function Z-score	-0.06 ± 0.85		-0.09 ± 0.76		0.500
ΔMemory Z-score	-0.13 ± 0.90		-0.06 ± 0.89		0.455
Brain atrophy rate	0.96 ± 0.67		0.73 ± 0.61		0.003*


* P-value < 0.05; CDR-global, Clinical Dementia Rating scale (CDR) global score; CDR-SOB, CDR sum of boxes score; Δ, cognitive change (follow-up minus baseline); ↓, decreased (improvement); ↔, unchanged; ↑, increased (cognitive decline).

Table 3 showed the results of interaction analysis of aspirin use on the effect of B vitamins in cognitive function and whole brain atrophy rate. Aspirin use had significant interaction effect in ΔCDR-global and ΔCDR-SOB (Beta = 0.993, P = 0.038, and Beta = 0.583, P = 0.009, respectively), but not in whole brain atrophy rate (Beta = 0.192, P = 0.276) or in memory or executive function Z-scores. Interaction analysis was also performed to examine the potential influence of baseline serum homocysteine on the effects of B vitamins in cognitive function and brain atrophy (data not shown). Baseline homocysteine concentration did not have significant interaction effects on ΔCDR-global, ΔCDR-SOB and whole brain atrophy rates (P = 0.628, 0.164 and 0.206, respectively).

**Table 3:** Interaction analysis† of aspirin use and B vitamins in cognitive change and whole brain atrophy rate

	**B vitamins**		**Aspirin**		**Interaction**	
	**Beta**	**P-value**	**Beta**	**P-value**	**Beta**	**P-value**
ΔCDR-global	−0.345	0.387	−0.512	0.142	0.993	0.038*
ΔCDR-SOB	−0.330	0.730	−0.246	0.694	0.583	0.009*
ΔExecutive function Z-score	−0.013	0.723	−0.006	0.902	0.033	0.846
ΔMemory Z-score	0.088	0.362	−0.011	0.893	0.005	0.981
Brain atrophy rate	−0.088	0.038*	0.192	0.289	0.192	0.276


† Beta and P values of the effects of B vitamins, aspirin and their interaction in multiple linear regression model with age, sex, education years, study site, body mass index (BMI), smoking status, and history of diabetes and stroke and serum homocysteine as covariates ; * P-value < 0.05; CDR-global, Clinical Dementia Rating scale (CDR) global score; CDR-SOB, CDR sum of boxes score; Δ, cognitive change (follow-up minus baseline).

Table 4 showed the group differences in changes in cognitive function and whole brain atrophy rate among aspirin users and non-users. Among aspirin non-users, as compared with placebo group subjects, active group subjects had significantly more favourable ΔCDR-global (Beta = -0.604, P = 0.019), ΔCDR-SOB (Beta = −0.223, P = 0.057) and significantly lower whole brain atrophy rate (Beta = −0.292, P = 0.001). Among aspirin users, none of these differences were significant, and ΔCDR-SOB tended to increase with B vitamins (P = 0.054). Among placebo group subjects, aspirin users had significantly less increase in CDR-SOB than aspirin non-users. (P = 0.019).

**Table 4:** Subgroup analysis of the effects of B vitamins in cognitive function and whole brain atrophy rate in aspirin non-users and users

	**Aspirin non-user**				**Aspirin user**				**P-value**			
	**Placebo (N = 191)**		**Active (N = 200)**		**Placebo (N = 83)**		**Active (N = 71)**		**P1**	**P2**	**P3**	**P4**
ΔCDR-global	↓	21 (14.1%)	↓	36 (22.9%)	↓	13 (19.4%)	↓	8 (13.6%)	0.019*	0.191	0.099	0.221
	↔	107 (71.8%)	↔	109 (69.4%)	↔	50 (74.6%)	↔	46 (78.0%)				
	↑	21 (14.1%)	↑	12 (7.6%)	↑	4 (6.0%)	↑	5 (8.5%)				
ΔCDR-SOB	0.34 ± 1.15		0.08 ± 0.94		−0.02 ± 0.95		0.30 ± 1.11		0.057	0.019*	0.067	0.054
ΔExecutive function Z-score	−0.06 ± 0.88		−0.10 ± 0.75		−0.06 ± 0.77		−0.07 ± 0.78		0.538	0.940	0.868	0.893
ΔMemory Z-score	−0.12 ± 0.91		−0.05 ± 0.88		−0.16 ± 0.86		−0.09 ± 0.93		0.595	0.698	0.991	0.682
Brain atrophy rate	0.95 ± 0.69		0.68 ± 0.56		0.99 ± 0.62		0.89 ± 0.74		0.001*	0.733	0.083	0.500


* P-value < 0.05, P1: «aspirin non-user + placebo» vs. «aspirin non-user + active», P2: «aspirin non-user + placebo» vs. «aspirin user + placebo», P3: «aspirin non-user + active» vs. «aspirin user + active», P4: «aspirin user + placebo» vs. «aspirin user + active»; CDR-global, Clinical Dementia Rating scale (CDR) global score; CDR-SOB, CDR sum of boxes score; Δ, cognitive change: follow-up minus baseline; ↓, decreased (improvement); ↔, unchanged; ↑, increased (cognitive decline).

## Discussion

Using pooled data in the VITACOG and HK trials, we found that homocysteine-lowering by B vitamins consistently lowered whole brain atrophy rates in older people with MCI, though the modest effect in cognitive function observed in the VITACOG was not replicated in the HK trial. More interestingly, we found a significant negative interaction effect of aspirin on the effects of B vitamins in global cognitive functioning. B vitamins significantly improved cognitive functioning and slowed brain atrophy among those were not taking aspirin, but not in those who were.

e VITACOG and HK trials shared a similar design (7, 8, 9). But there were significant differences. Firstly, there were some differences in clinical characteristics of subjects in the two trials. The UK subjects had more education and lower prevalence of diabetes mellitus, though they had higher prevalence of aspirin use, stroke and ever-smoking (Table S1). Overall, the VITACOG subjects had significantly higher whole brain atrophy rate than HK trial subjects (0.92 ± 0.67% versus 0.71 ± 0.59%; P = 0.010). Secondly, the VITACOG trial used cyanocobalamin 500 µg, folic acid 800 µg and vitamin B6 20 mg once daily, whereas in the HK trial the dose of folic acid was limited to 400 µg and vitamin B6 was not given. Nevertheless, both B-vitamin formulations lowered serum homocysteine by about one-third (7, 8, 9), and previous trials did not suggest significant additive effects of vitamin B6 in combination with vitamin B12 and folic acid, in either the extent of homocysteine-lowering or cognitive outcomes (15, 26).

The pooled data analysis reaffirmed the significant reduction in brain atrophy with B vitamins, which was initially found in the VITACOG trial (7). This is consistent with the notion that homocysteine contributes to AD. However, the pooled data analysis did not show significant benefit in cognitive function or functioning, even though the VITACOG trial showed a modest benefit in executive function (clock drawing test) with B vitamins, and clear benefit in other cognitive tests and CDR in those with higher serum homocysteine [8]. Unfortunately, the HK trial did not include clock drawing test and we had to use category fluency test for executive function in this analysis.

The most important finding of this analysis is the negative interaction effect of aspirin on the cognitive effect of B vitamins. This negative interaction effect of aspirin was first reported in the HK trial (9). This pooled analysis further showed that this negative interaction effect was significant for global cognitive functioning in MCI subjects, and the interaction effect was independent of comorbidities (e.g., stroke, diabetes mellitus). Although the interaction term did not reach significance, it is noteworthy that B vitamins slowed brain atrophy very significantly in aspirin non-users, but not significantly so in aspirin-users. This highlights the importance of subgroup analysis in identifying subject groups who may benefit from the intervention in negative trials.

Out of the three B vitamins, folic acid was the one which was most likely to have interacted with aspirin because vitamin B6 was not used in the HK trial and the post doc analysis of our other randomized trial of vitamin B12 supplementation in older people (22) did not find any interaction effect of aspirin use (unpublished data).

A possible explanation for the potential negative interaction effect of aspirin on the cognitive benefit from folic acid is that aspirin may have antifolate effects (27, 28, 29). For example, oral administration of 650 mg aspirin every 4 hours for three days induced a significant but reversible fall in total and bound serum folate and a small rise in urinary folate excretion (27). Besides, aspirin in vitro also displaced significant amounts of bound serum folate in a dose related manner (27, 28). When using aspirin (5–8 g daily) to treat inflammation, Baggott et al. noticed an inhibition of folate-dependent dihydrofolate reductase (DHFR) enzyme (29). However, it is uncertain if low-dose aspirin, which is much more commonly used, has similar anti-folate effects.

Folic acid is a synthetic form of folate which requires metabolism into dihydrofolate and then to tetrahydrofolate by DHFR (30), the first step being slow and rate limiting. DHFR expression in human liver is also low and variable (30). Aspirin has been shown to inhibit the expression of E2F-1 which regulates DHFR expression in human lung cancer cells (31). It is possible that aspirin may inhibit the upregulation of DHFR in the liver, which normally occurs with folic acid supplementation (32), resulting in higher concentrations of circulating unmetabolized folic acid (UMFA).

There have been concerns about the potential harm of UMFA (33, 34, 35, 36), one of which is that UMFA may impair the active transport of active folates into the brain (37, 38). In addition, salicylic acid, the hydrolysate of aspirin, was reported to be a low-affinity inhibitor of reduced folate carrier-1 (RFC-1) (39), which plays a major role in delivery of reduced folate into the brain (40, 41, 42). Therefore, inhibition of RFC-1 by salicylate at the level of blood brain barrier may result in cerebral folate deficiency, leading to cognitive impairment.

Another possible explanation for aspirin/folate interaction is that folate may interfere with the anti-inflammatory effects of aspirin. In a 3×2 randomized trial of aspirin (81 or 325 mg, placebo) and folic acid (1 mg, placebo) to prevent recurrence of colonic adenoma (43), it was found that higher dose aspirin (325 mg) reduced serum C reactive protein, but this was abrogated by folic acid (43). Although low-dose aspirin did not lower serum C reactive protein significantly in this trial, it has been shown to have anti-inflammatory effects (44). Whether folate can abrogate the anti-inflammatory effect of low-dose aspirin has not been examined.

Aspirin is most commonly used in low doses for its anti-platelet actions which help to prevent myocardial infarction and stroke. It is noteworthy that there have been suggestions of an aspirin/folic acid negative interaction in stroke prevention. Most large randomized placebo-controlled trials of folic acid with or without other B vitamins for prevention of cardiovascular outcomes have shown negative results (10, 12, 13, 15). But a post-hoc analysis of the HOPE-2 trial suggested a larger benefit of B-vitamin therapy for stroke prevention among those not receiving antiplatelet drugs (mainly aspirin), and a post-hoc analysis of the VITATOPS trial found that B vitamins prevented further strokes in stroke patients only in those not taking aspirin (45, 46). Moreover, the few trials which showed benefits of folic acid in stroke prevention involved subjects with low or no usage of aspirin (11, 14, 47). This apparent aspirin/folic acid negative interaction in stroke incidence is yet to be explained. It is tempting to speculate that folic acid impairs the anti-platelet actions as well as the anti-inflammatory effects of aspirin.

The anti-platelet effects of aspirin are due to its inhibition of prostaglandin synthesis by inhibiting cyclooxygenase (COX)-1 and COX-2 enzymes. Both COX-1 and COX-2 activities have been implicated in AD pathology (48, 49, 50). Biological mechanisms include inhibition of neuroinflammatory responses (51), reduction in amyloid-β formation (52) and tau phosphorylation (53). Epidemiological studies have consistently shown that aspirin users are less likely to have AD than nonusers (54, 55). Although a large trial of low-dose aspirin in older people did not find lower incidence of dementia over 5 years (56), it remains possible that aspirin may slow cognitive decline in older people with MCI. This was borne out in our analysis in that among placebo group subjects in both trials of older people with MCI, aspirin users had significantly less cognitive decline than aspirin non-users. It is therefore possible that folate interferes with the neuroprotective effect of aspirin in older people with MCI.

After excluding aspirin users, this study showed convincing evidence that B vitamins either improved or prevented deterioration in global cognitive functioning as well as reducing rates of brain atrophy in older people with MCI. Whether B vitamins can prevent or delay onset of dementia in this high-risk group requires a larger and longer trial.

In contrast with the VITACOG trial, this pooled analysis did not show a significant interaction effect of baseline serum homocysteine on the response in CDR and brain atrophy rate with B vitamins. A possible explanation is that the serum homocysteine concentrations were generally higher in the subjects in the HK trial. As a result, the post supplement serum homocysteine concentrations were still above the safety level for cognitive impairment or neurodegeneration. It is noteworthy that HK trial subjects had lower whole brain atrophy rate than the VITACOG trial despite having higher serum homocysteine. This suggested that the MCI subjects in HK trial were in an earlier phase of neurodegeneration.

This study had limitations. Firstly, the two trials were significantly different in some important subjects' characteristics e.g. ethnicity, education level, vascular burden and baseline serum homocysteine. Secondly, the B-vitamin formulations used in the trials were different. Thirdly, the cognitive tests in common were limited; category fluency test is not a specific test for executive function, it being influenced by semantic memory; HVLT and ISLT were slightly different, though we overcame this by transforming the data into z scores in the respective trial population. Fourthly, serum homocysteine in the VITACOG trial were taken non-fasting while those in the HK trial were taken after fasting overnight, but the estimated difference in serum homocysteine taken with or without fasting has been found to be around 10% [57]. Lastly, aspirin use was not randomly assigned, the possibility of bias by indication cannot be excluded by adjustments.

## Conclusions

In conclusion, our pooled analysis suggested a significant drug-nutrient interaction between aspirin and B vitamins in older people with MCI. B vitamins improved global cognitive functioning and slowed brain atrophy significantly in those not taking aspirin, but not in aspirin users. The negative interaction is most likely to lie between aspirin and folic acid. Further investigations on the mechanisms underlying this interaction may lead to more effective use of B vitamins in the prevention of dementia.

## Electronic supplementary material

Effectiveness of B vitamins and their interactions with aspirin in improving cognitive functioning in older people with mild cognitive impairment: pooled post-hoc analyses of two randomized trials
