# Supplementary material for: Effectiveness of B Vitamins and Their Interactions with Aspirin in Improving Cognitive Functioning in Older People with Mild Cognitive Impairment: Pooled Post-Hoc Analyses of Two Randomized Trials
Source: J Nutr Health Aging. 2021 Nov 24;25(10):1154–60. doi: 10.1007/s12603-021-1708-1 (PMC12275523; doi:10.1007/s12603-021-1708-1)
Supplement: Supplementary file 1 — Effectiveness of B vitamins and their interactions with aspirin in improving cognitive functioning in older people with mild cognitive impairment: pooled post-hoc analyses of two randomized trials [file mmc1.docx]

**Effectiveness of B vitamins and their interactions with aspirin in improving cognitive functioning in older people with** **mild cognitive impairment: pooled post-hoc analyses of two randomized trials**

**Supplementary material**

**Table S1.** Comparison of baseline characteristics of subjects in VITACOG and HK trials

|  | UK  (N =266) | HK  (N =279) | P-value |
| --- | --- | --- | --- |
| Age, years | 76.8 ± 4.9 | 77.4 ± 5.3 | 0.152 |
| Female, n (%) | 170 (63.9%) | 113 (40.5%) | < 0.001^*^ |
| Years of education | 14.5 ± 3.4 | 6.6 ± 4.8 | < 0.001^*^ |
| BMI, kg/m^2^ | 26.0 ± 4.0 | 24.7 ± 3.3 | < 0.001^*^ |
| Ever smoking, n (%) | 125 (47.3%) | 83 (29.7%) | < 0.001^*^ |
| Aspirin user, n (%) | 90 (33.8%) | 64 (22.9%) | 0.005^*^ |
| HBP, n (%) | 194 (72.9%) | 182 (65.2%) | 0.052 |
| DM, n (%) | 14 (5.3%) | 87 (31.2%) | < 0.001^*^ |
| Stroke, n (%) | 32 (12.2%) | 18 (6.5%) | 0.021^*^ |
| Hb, g/dL | 13.8 ± 1.2 | 13.6 ± 1.3 | 0.080 |
| MCV, fL | 92.5 ± 4.5 | 89.9 ± 7.6 | < 0.001^*^ |
| TG ^#^, g/L | 1.3 (0.9, 1.7) | 1.2 (0.9, 1.6) | 0.231 |
| TC, mmol/L | 5.5 ± 1.1 | 4.7 ± 1.0 | < 0.001^*^ |
| Cr, μmol/L | 96.9 ± 16.6 | 89.2 ± 24.7 | < 0.001^*^ |
| Homocysteine ^#^, μmol/L | 11.4 (9.6, 13.5) | 16.6 (14.1, 19.8) | < 0.001^*^ |
| Folate ^#^, nmol/L | 21.7 (13.2, 37.4) | 27.8 (21.9, 33.7) | < 0.001^*^ |
| HoloTC ^#^, pmol/L | 67.0 (50.0, 91.0) | 85.9 (58.5, 116) | < 0.001^*^ |
| CDR-global = 0, n (%) | 79 (29.7%) | 40 (14.3%) | < 0.001^*^ |
| Drop out n (%) | 43 (16.2%) | 41 (14.7%) | 0.635 |

Data were shown as “mean ± SD”, “median (Q1, Q3)” or “n (%)” as appropriate;

^#^ Use log-transformed data for comparison;

^*^ P-value < 0.05;

BMI, body mass index; HBP, high blood pressure; DM, diabetes mellitus; Hb, haemoglobin; MCV, mean corpuscular volume; TG, triglycerides; TC, total cholesterol; Cr, creatinine; HoloTC, holotranscobalamin (active vitamin B_12_); CDR-global, Clinical Dementia Rating scale (CDR) global score
